# The genetic landscape and classification of infantile epileptic spasms syndrome requiring surgery due to suspected focal brain malformations

**DOI:** 10.1093/braincomms/fcaf034

**Published:** 2025-01-25

**Authors:** Matthew Coleman, Min Wang, Penny Snell, Wei Shern Lee, Colleen D'Arcy, Cristina Mignone, Kate Pope, Greta Gillies, Wirginia Maixner, Alison Wray, A Simon Harvey, Cas Simons, Richard J Leventer, Sarah E M Stephenson, Paul J Lockhart, Katherine B Howell

**Affiliations:** Murdoch Children’s Research Institute, Parkville, Victoria 3052, Australia; Department of Paediatrics, The University of Melbourne, Melbourne, Victoria 3052, Australia; Murdoch Children’s Research Institute, Parkville, Victoria 3052, Australia; Murdoch Children’s Research Institute, Parkville, Victoria 3052, Australia; Murdoch Children’s Research Institute, Parkville, Victoria 3052, Australia; Department of Paediatrics, The University of Melbourne, Melbourne, Victoria 3052, Australia; Department of Anatomical Pathology, The Royal Children’s Hospital, Parkville, Victoria 3052, Australia; Department of Medical Imaging, The Royal Children's Hospital, Parkville, Victoria 3052, Australia; Murdoch Children’s Research Institute, Parkville, Victoria 3052, Australia; Murdoch Children’s Research Institute, Parkville, Victoria 3052, Australia; Department of Neurosurgery, The Royal Children’s Hospital, Parkville, Victoria 3052, Australia; Department of Neurosurgery, The Royal Children’s Hospital, Parkville, Victoria 3052, Australia; Murdoch Children’s Research Institute, Parkville, Victoria 3052, Australia; Department of Paediatrics, The University of Melbourne, Melbourne, Victoria 3052, Australia; Department of Neurology, The Royal Children's Hospital, Parkville, Victoria 3052, Australia; Murdoch Children’s Research Institute, Parkville, Victoria 3052, Australia; Centre for Population Genomics, Garvin Institute of Medical Research, and UNSW Sydney, Sydney, New South Wales 2010, Australia; Murdoch Children’s Research Institute, Parkville, Victoria 3052, Australia; Department of Neurology, The Royal Children's Hospital, Parkville, Victoria 3052, Australia; Murdoch Children’s Research Institute, Parkville, Victoria 3052, Australia; Department of Paediatrics, The University of Melbourne, Melbourne, Victoria 3052, Australia; Murdoch Children’s Research Institute, Parkville, Victoria 3052, Australia; Department of Paediatrics, The University of Melbourne, Melbourne, Victoria 3052, Australia; Murdoch Children’s Research Institute, Parkville, Victoria 3052, Australia; Department of Neurology, The Royal Children's Hospital, Parkville, Victoria 3052, Australia

**Keywords:** infantile epileptic spasms syndrome, focal brain malformation, mild malformation of cortical development with oligodendroglial hyperplasia in epilepsy, *SLC35A2*

## Abstract

Infantile epileptic spasms syndrome is a severe epilepsy of infancy that is often associated with focal malformations of cortical development. This study aimed to elucidate the genetic landscape and histopathologic aetiologies of infantile epileptic spasms syndrome due to focal malformations of cortical development requiring surgery. Fifty-nine children with a history of infantile epileptic spasms syndrome and focal malformations of cortical development on MRI were studied. Genetic testing of resected brain tissue was performed by high-coverage targeted panel sequencing or exome sequencing. Histopathology and MRI were reviewed, and integrated clinico-pathological diagnoses were established. A genetic diagnosis was achieved in 47 children (80% of cohort). Germline pathogenic variants were identified in 27/59 (46%) children, in *TSC2* (x19), *DEPDC5* (x2), *CDKL5* (x2), *NPRL3* (x1), *FGFR1* (x1), *TSC1* (x1), and one child with both a *TUBB2A*/*TUBB2B* deletion and a pathogenic variant in *COL4A1* (x1). Pathogenic brain somatic variants were identified in 21/59 (36%) children, in *SLC35A2* (x9), *PIK3CA* (x3), *AKT3* (x2), *TSC2* (x2), *MTOR* (x2), *OFD1* (x1), *TSC1* (x1) and *DEPDC5* (x1). One child had ‘two-hit’ diagnosis, with both germline and somatic pathogenic *DEPDC5* variants in trans. Multimodal data integration resulted in clinical diagnostic reclassifications in 24% of children, emphasizing the importance of combining genetic, histopathologic and imaging findings. Mammalian target of rapamycin pathway variants were identified in most children with tuberous sclerosis or focal cortical dysplasia type II. All nine children with somatic *SLC35A2* variants in brain were reclassified to mild malformation of cortical development with oligodendroglial hyperplasia in epilepsy. Somatic mosaicism was a major cause of focal cortical dysplasia type II/hemimegalencephaly (81%) and mild malformation of cortical development with oligodendroglial hyperplasia (100%). The genetic landscape of infantile epileptic spasms syndrome due to focal malformations comprises germline and somatic variants in a range of genes, with mTORopathies and *SLC35A2*-related mild malformation of cortical development with oligodendroglial hyperplasia being the major causes. Multimodal data integration incorporating genetic data aids in optimizing diagnostic pathways and can guide surgical decision-making and inform future research and therapeutic interventions.

## Teaser

Coleman *et al*. report a comprehensive study of the genetic, imaging and histopathologic findings in 59 children with infantile epileptic spasms syndrome due to focal brain malformations requiring epilepsy surgery. They identify the genetic basis in 80% of children and show that brain somatic variants in *SLC35A2* are a major contributor.

## Graphical Abstract

**graphical abstract:**
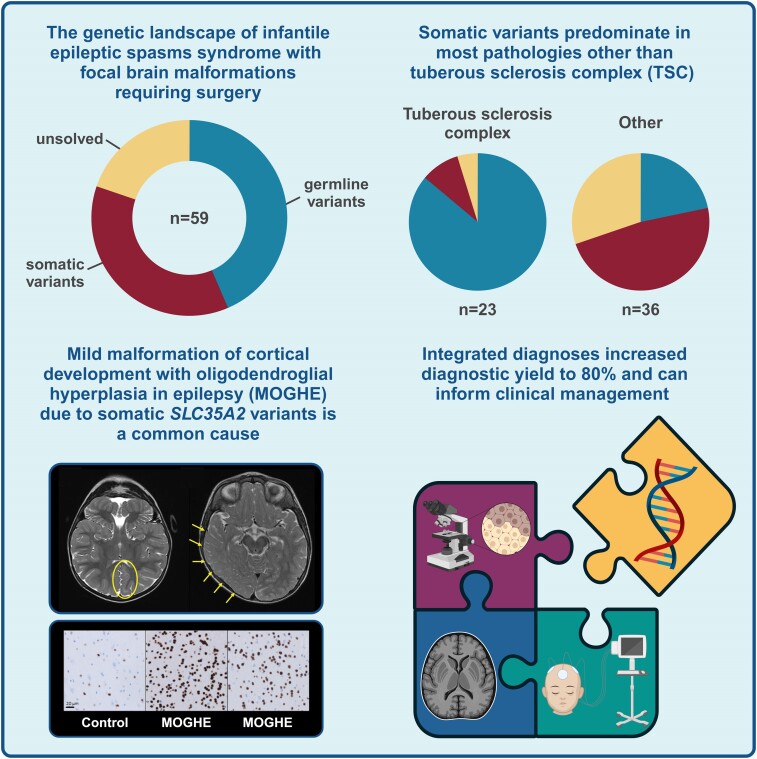

## Introduction

Infantile epileptic spasms syndrome (IESS) is the most common severe epilepsy of infancy, with an incidence of ∼1:3000 live births.^1-3^ While many different brain pathologies cause IESS, focal structural abnormalities, including focal cortical dysplasia (FCD) and tuberous sclerosis complex (TSC) and other focal malformations of cortical development (MCD), are the most common aetiologies.^4,5^ We previously reported that focal MCD are underdiagnosed in infants with IESS, being difficult to identify on brain imaging during infancy.^6^ In our population-based cohort study of infants with severe epilepsy, 18% with IESS had focal MCD, compared with 0.5–2.3% in previously reported cohorts.^7-9^

Seizure and developmental outcomes in IESS are poor but can be improved with prompt, effective treatment of spasms.^10^ Optimal treatment strategies are influenced by knowledge of the underlying cause. In particular, IESS due to focal MCD are frequently resistant to antiseizure medications and often require epilepsy surgery for seizure control and to halt progressive developmental impacts.^2,10^

In recent years, advances in sequencing technologies have enabled comprehensive genomic analyses of surgical tissue from focal MCD causing epilepsy, increasing understanding of the causative genes and highlighting somatic mosaicism as a major genetic mechanism in these conditions.^11-13^ Strong phenotype–genotype correlations are now recognized in several MCD subtypes, in particular, between the PIK3/AKT/mTOR and GATOR1 complex signalling pathways and TSC and FCD type 2 (FCDII; collectively termed ‘mTORopathies’)^11,14,15^ and between the Solute Carrier Family 35 Member A2 (*SLC35A2)* gene and a more recently-recognized clinical entity, mild malformation of cortical development with oligodendroglial hyperplasia in epilepsy (MOGHE).^4,16^

Most studies of the genetic basis of focal MCD have focused on cohorts grouped by histopathologic diagnosis rather than the epilepsy syndrome.^5,11^ Although IESS are reported in individuals with focal MCD due to the aforementioned genes, it is not clear whether the genetic landscape of focal MCD causing IESS differs from that of focal MCD causing other epilepsies. Understanding the genetic basis of IESS due to focal MCD may aid in optimizing diagnostic pathways to expedite identification of a surgically-remediable cause of IESS, expand understanding of the pathogenesis of IESS and inform identification or development of treatment alternatives to surgery.

In this study, we investigated the genetic landscape and histopathologic aetiologies of epilepsy due to focal MCD in a cohort of 59 children with IESS who underwent epilepsy surgery.

## Materials and methods

### Standard protocol approvals, registrations and patient consents

The Human Research Ethics Committee of The Royal Children’s Hospital, Melbourne, Australia, approved this study (HREC #29077, #38095 and #32288). Written informed consent was obtained from a parent or legal guardian of each child.

### Study cohort

Children with IESS (onset < age 18 months) who underwent epilepsy surgery for drug-resistant seizures at the Royal Children’s Hospital, Melbourne, Australia, during 2011–2022 were identified from the Royal Children’s Hospital Children’s Epilepsy Surgery Program database and two research databases at the Murdoch Children’s Research Institute (Neurogenetics Laboratory epilepsy surgery brain tissue bank database, and Severe Epilepsy of Infancy Study database). We excluded children with acquired causes of IESS (e.g. stroke, infection) and children for whom brain tissue was unavailable. We identified 62 eligible participants, and consent for research genomic testing on brain tissue specimens was obtained for 59/62 (95%).

### Clinical data collection and review

Clinical features [including age at seizure onset, seizure types, age at surgery, type of surgery, seizure outcome (Engel class)], and results of EEG, MRI, clinical genetic testing, and histopathology were extracted from medical records.

### Tissue specimen collection

Brain tissue specimens were collected at the time of surgery and stored as both fresh frozen specimens and formalin-fixed paraffin-embedded tissue blocks.

### Genetic testing

Genetic testing was performed on genomic DNA extracted from resected brain specimens for all participants (*n* = 59).

#### DNA extraction

Genomic DNA was extracted from fresh frozen brain tissue using a Qiagen AllPrep DNA/RNA Mini Kit (Valencia, CA, USA, no. 80204) according to the manufacturer’s instructions. Saliva and blood samples were extracted using NucleoBond CB20 and CB100 respectively (Macherey-Nagel, Düren, Germany, no. 740507 and no. 740508).

#### Sequencing

##### Haloplex^HS^ targeted panel sequencing

Twenty-seven children with TSC or FCDII, expected to carry variants in PI3K-AKT-mTOR pathway or GATOR1 complex genes, underwent targeted panel sequencing using a custom-designed HaloPlex^HS^ gene panel as previously described.^17,18^ Children with no genetic basis identified following Haloplex^HS^ analysis underwent exome sequencing (ES).

##### Exome Sequencing

ES was performed on brain-derived DNA from 41 children (9 unsolved by prior HaloPlex^HS^ gene panel, 32 with no prior testing on brain tissue) with either TWIST (24) or CREv2 library preparation (17) using an Illumina NovaSeq6000 sequencer (2 × 150 bp) at a 400× target read depth. If unresolved following this singleton ES analysis, trio ES analysis was undertaken using blood-derived gDNA from unaffected parents.

#### Variant analysis

##### Single nucleotide variant analysis

Germline and somatic candidate variants were detected in HaloPlex^HS^ targeted panel sequencing data using SureCall software (Agilent), in which alignment and variant calling were performed automatically using user-defined parameters. ES data were analysed using HaplotypeCaller (joint-calling) and Mutect2 (tumour-only mode), respectively, following the GATK^19^ Best Practice Workflows (v4.2.0.0, Broad Institute). Variants were prioritized using seqr (v.0.3.0, https://github.com/broadinstitute/seqr) with the following inclusion criteria: (1) within a focal brain malformation gene list from PanelApp Australia^20^ and in-house lists of confirmed and candidate genes for MCD and/or IESS (2021 genes), (2) coding/nonsynonymous, an insertion/deletion, or close proximity to a splice site, (3) population allele frequency ≤0.001 in dominant genes or ≤0.01 in recessive genes and (4) predicted to be damaging by two or more *in silico* tools (CADD,^21^ REVEL,^22^ PrimateAI,^23^ MPC,^24^ SpliceAI,^25^ EIGEN,^26^ PolyPhen,^27^ SIFT,^28^ MutTaster,^29^ FATHMM,^30^ MetaSVM^31^).

##### Copy number variant analysis

CNVs were identified from ES data using C to Go translator, CxGo.^32^ Candidate CNVs were filtered as being *de novo* and predicted damaging by one or more *in silico* tools (ed, xhmm, cdx).

#### Candidate variant validation

Novel variants or variants of uncertain significance were validated via Sanger sequencing segregation or droplet digital PCR validation. No independent confirmation was performed if the variant had been reported as pathogenic (P) or likely pathogenic (LP) in ClinVar previously.

##### Sanger sequencing

Germline variants [variant allele frequency (VAF) > 40%] were validated and segregated using PCR and Sanger sequencing. Genetic variants were amplified using gene-specific primers (oligonucleotide sequences available on request) designed to the reference human gene transcripts. Sanger sequencing products were sequenced at the Victorian Clinical Genetics Services on a 3730xl DNA Analyzer (Thermo Fisher Scientific). Sanger trace data were aligned and analysed using CodonCode Aligner v9.0.1 (CodonCode Corporation).

##### Droplet digital PCR

To validate somatic single nucleotide variant, we performed droplet digital PCR on paired blood- and brain-derived gDNA samples. We used Custom TaqMan SNP Genotyping Assay (Thermo Fisher Scientific, Waltham, MA, USA) according to the manufacturer’s protocol (sequences available upon request).

##### High-density chromosomal microarray

CNVs identified through CxGO analysis were validated via clinical testing with Infinium Global Diversity Array-8 if <1Mb in size or only detected by one *in silico* method.

#### Variant classification

Candidate variants were classified according to American College of Medical Genetics and Genomics guidelines.

### Review of histopathology and brain imaging

Children in whom genetic findings and clinical histopathological findings were discordant, or no genetic diagnosis was identified, underwent blinded review of histopathology and MRI. Histopathology review was performed on newly stained brain tissue specimens closest to the most prominent area of abnormality on MRI. Immunohistochemical staining was performed according to established protocols using antibodies directed against OLIG2 (CellMarque, no. EP112, dilution 1:1000), Phospho-S6 Ribosomal Protein (Ser235/236) (pS6) (Cell Signaling, no. 4858P, dilution 1:100), NeuN (Millipore, no. MABN140, dilution 1:100) and MAP2 (Sigma, no. M1406, dilution 1:500) and counter stained with haematoxylin. A neuropathologist (C.D.) reviewed the specimens and classified findings according to the ILAE Diagnostic Methods Commission criteria.^4,5^

MRI brain scans were reviewed by a neuroradiologist (C.M.). The location and extent of abnormalities and a description of the cortical (e.g. thickness, signal), corticomedullary (e.g. differentiation, signal), white matter (e.g. myelination, volume, signal) and subcortical features were documented. An overall impression of likely underlying pathology was provided, based on published reports of the typical imaging features of particular focal MCD (e.g. MOGHE subtype 1 = linear hyperintensity at the grey-white matter junction, subtype 2 = blurred grey-white matter junction due to increased T2 signal in the white matter, FCDII/HME = variable features, which may include thick cortex with abnormal signal, abnormal gyration, abnormal white matter signal, larger hemisphere/quadrant, FCDI = variable features, may be subtle, and can include smaller hemisphere/quadrant, abnormal gyration, abnormal white matter signal).^33,34^

### Final diagnostic classification

Genetic, histopathologic and MRI data were combined to produce an integrated clinico-pathological diagnosis.^2,4^

### Statistical analysis

We used descriptive statistics to compare relative proportions. The histopathologic diagnoses of FCDI, mMCD and no FCD identified were analysed as a single group. Differences between groups were compared using the χ^2^ statistic or Fisher’s exact test, for categorical data, and *t*-tests of median values, for continuous data.

## Results

### Clinical characteristics

Twenty-six of the 59 children (44%) were female (Supplementary Table 1). Median age at seizure onset was 4 months (range 0–16 months, IQR 1.5–6.5). Fifty-one children (88%) had other seizure types in addition to epileptic spasms. Thirty patients (51%) had hypsarrhythmia or modified hypsarrhythmia at some point. All except one patient had focal features to their EEG, either as a focal abnormality or as a focal predominance within a bilaterally abnormal EEG (e.g. modified hypsarrhythmia with prominent focal slowing). The location of focal EEG features was typically concordant with the imaging abnormalities. The indication for epilepsy surgery was drug-resistant epileptic spasms in 15, drug-resistant focal seizures in 24 and drug-resistant seizures of multiple types in 20 children. Eight individuals with TSC were treated with oral mTOR inhibitors, for seizures or another TSC-related indication, typically after one or more epilepsy surgeries. No patients with FCDII received mTOR inhibitors and no patients with SLC35A2-MOGHE were treated with galactose supplementation. MRI abnormalities were unilateral in 32, bilateral in 4 and multifocal (unilateral or bilateral) in 23. Children underwent a total of 104 resective or disconnective surgeries (1–5 per patient, median 1), with 12 being hemispheric, 35 multilobar and 57 lobar/sublobar. The median age at first surgery was 2.4 years (range 0.1–16 years, IQR 1.3–3). Engel class I outcomes were achieved after the last surgery in 31 children (53%). The initial clinical diagnoses based on histopathology (+/− clinical features and MRI) were TSC 22/59 (37%), FCDII/HME 17/59 (28%), FCDI/mMCD/no FCD 18/59 (31%), dysembryoplastic neuroepithelial tumour 1/59 (2%) and complex MCD 1/59 (2%).

### Genetic diagnosis

**Figure 1:**
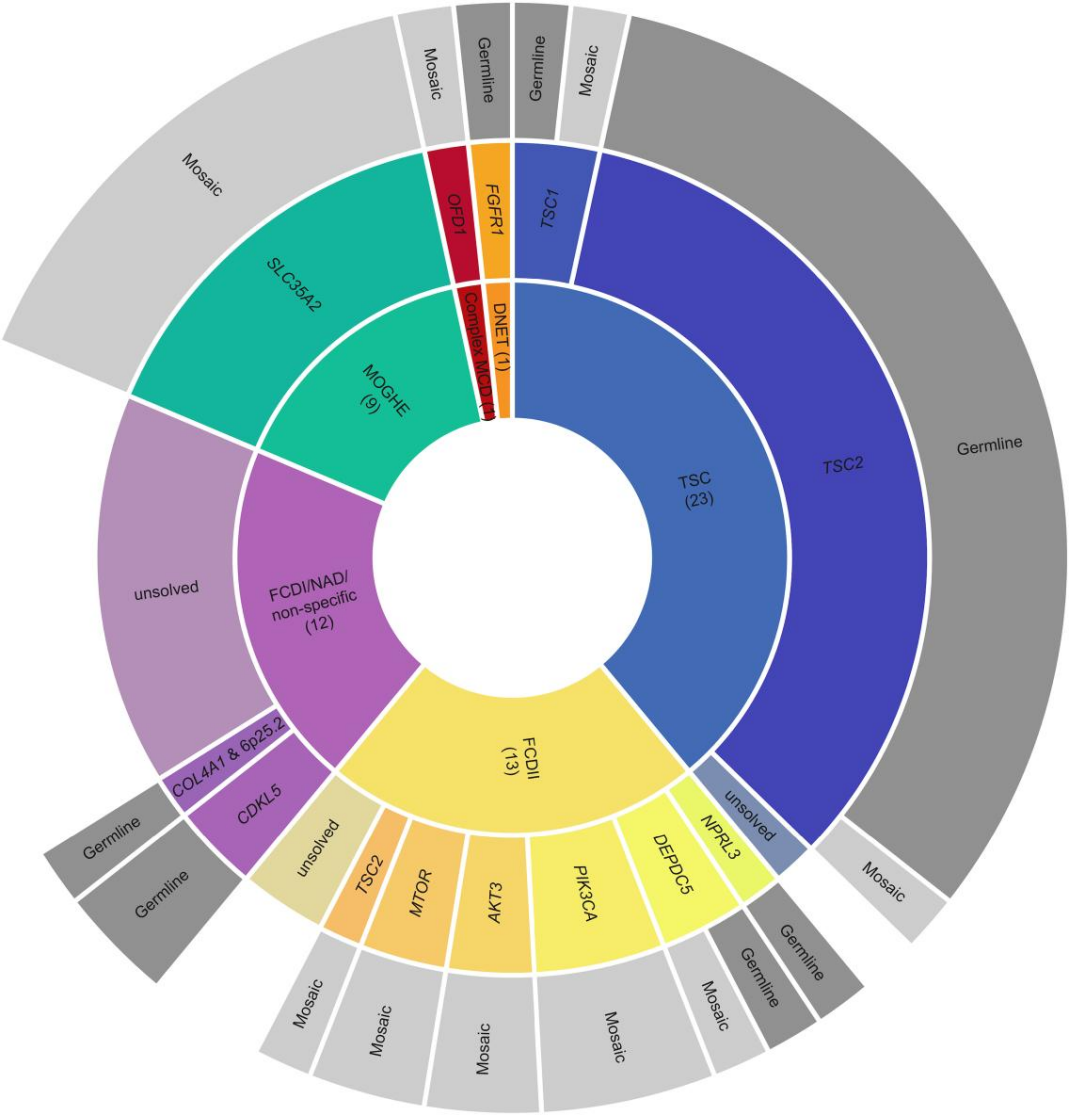
**Genetic findings by integrated diagnosis in 59 children with IESS.** The inner ring shows a pie graph of the MCD subtypes comprising this cohort. Number in brackets refers to number of children in each category. The middle ring lists the genes with pathogenic variants identified and associated with the respective phenotypes in this cohort. The outer ring denotes the inheritance (i.e. germline or mosaic) of these genetic variants in middle ring. If no genetic diagnosis was identified, genotype indicated as ‘unsolved’. Colours indicate MCD subtypes. Total *n* = 59.

A genetic cause for IESS was identified in 47/59 (80%) children (Supplementary Table 1, Fig. 1), four of which have been previously published.^6,35-37^ Twenty-eight pathogenic germline variants were identified in 27/59 (46%) children, in *TSC2* (*n* = 19), *DEPDC5* (2), *CDKL5* (2), *COL4A1* (1), 6p25.2 deletion (1), *NPRL3* (1), *FGFR1* (1) and *TSC1* (1). One individual had a blended phenotype as a result of a *COL4A1* variant and a 6p25.2 deletion that included *TUBB2A* and *TUBB2B*.

Pathogenic somatic variants were identified in brain tissue in 21/59 (36%) children in *SLC35A2* (9), *PIK3CA* (3), *AKT3* (2), *MTOR* (2), *TSC2* (2), *OFD1* (1), *TSC1* (1) and *DEPDC5* (1). VAFs of somatic variants ranged from 0.95 to 41% in brain tissue. None of the somatic variants identified in brain tissue were detected in blood via droplet digital PCR. One variant (*TSC2)* was detected in saliva in individual AA0936–01 at ∼10% via droplet digital PCR. One child had both a germline *DEPDC5* variant and a somatic *DEPDC5* variant.^35^ ‘Second hits’ were not detected in any other individuals in the cohort.

Genetic diagnosis was achieved in 21 (36%) children using Haloplex^HS^ gene panel sequencing and in 21 individuals (36%) using 400× ES. CNV analysis of 400 × ES data identified P/LP variants in an additional three (5%) children. The genetic cause was identified in four individuals through clinical testing in parallel to this study [*TSC2* CNV (2), *TSC2* single nucleotide variant (1) and *COL4A1* single nucleotide variant (1)], and other research genetic testing in one individual [germline *DEPDC5* variant (1) identified on Molecular Inversion Probe assay (MIPs) on peripheral tissue].^3^

These numbers include the two-hit *DEPDC5* diagnosis with the germline variant identified via HaloPlexHS and the somatic variant identified via ES seqr analysis; and the individual with a variant in *COL4A1* identified through ES seqr analysis and a 6p25.2 deletion identified via clinical CMA where both variants contribute to the phenotype. The genetic diagnosis was concordant with the clinical diagnosis (i.e. the clinical histopathology assessment and/or presurgical MRI) in 35/47 (75%) children with a genetic diagnosis.

### Histopathology and brain imaging review

**Figure 2:**
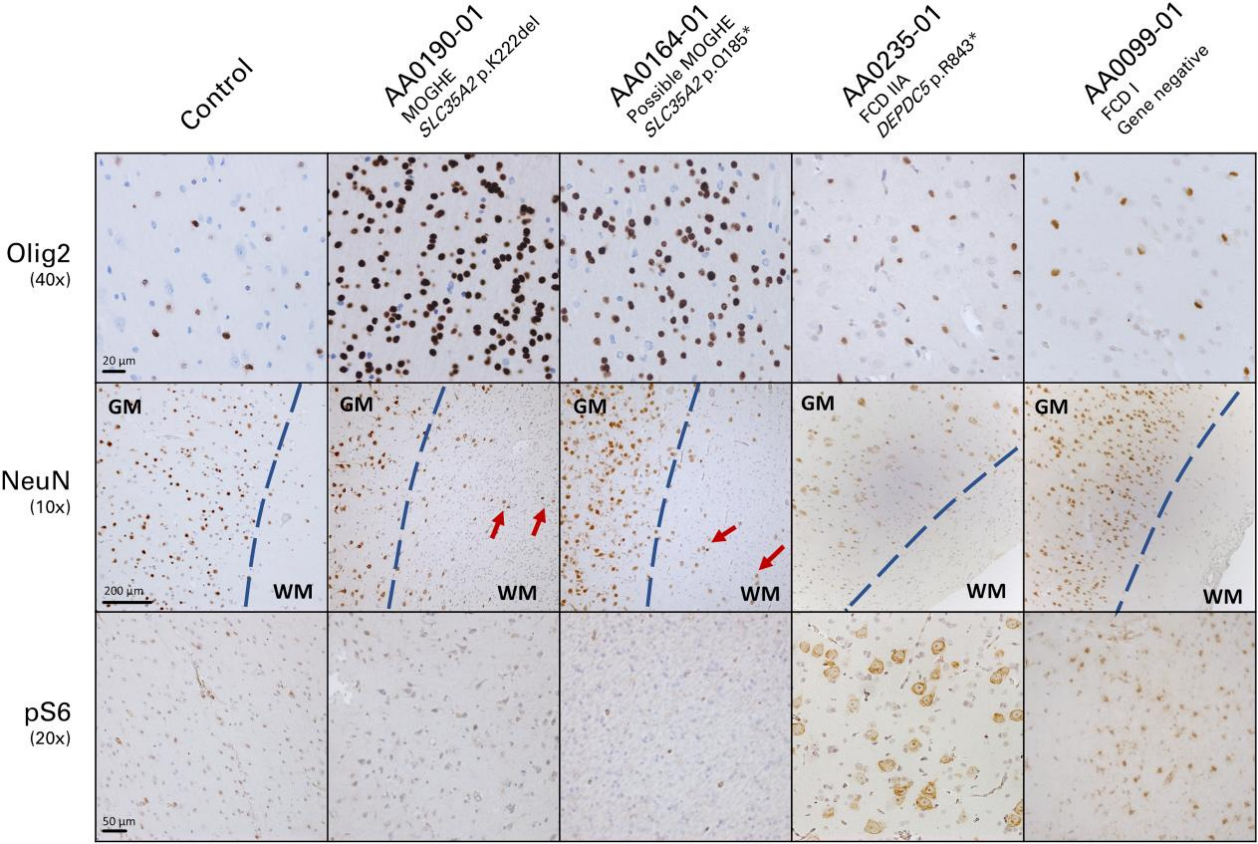
**Histopathological features in integrated diagnosis MOGHE cases.** Representative histopathology stains from an unaffected individual (Control), two MOGHE cases with *SLC35A2* mosaic variants in brain, one FCD IIA case with a heterozygous *DEPDC5* variant and one genetically unsolved FCD I case. Images of tissues have been chromogenically stained against anti-Olig2, anti-NeuN and anti-pS6. Blue dotted line indicates grey-white matter boundary. Red arrows indicate heterotopic neurons. 40×, 10× and 20× indicate magnification. Black bar indicates scale. GM , grey matter; WM, white matter.

Blinded review of histopathology and MRI was conducted for the 12 children with no genetic diagnosis, and 12 children in whom the genetic finding was discordant with the clinical diagnosis. One individual (AA0936-01), with a mosaic *TSC2* variant identified in saliva via concurrent clinical testing, which prompted reclassification from FCDIIB to TSC, did not undergo further review (Supplementary Table 1). Histopathologic review resulted in a revised histopathologic diagnosis in 13 individuals: nine to MOGHE [from FCDII (3) and FCDI/mMCD/no FCD (6)], two to FCDII (from FCDI/mMCD/no FCD) and two to FCDI/mMCD/no FCD (from FCDIIA; Supplementary Table 1, Fig. 2). All nine individuals reclassified to MOGHE had pathogenic somatic *SLC35A2* variants. *PIK3CA* variants were identified in the two individuals reclassified to FCDII. No genetic diagnoses were made in the two individuals reclassified to FCDI/mMCD/no FCD.

**Figure 3:**
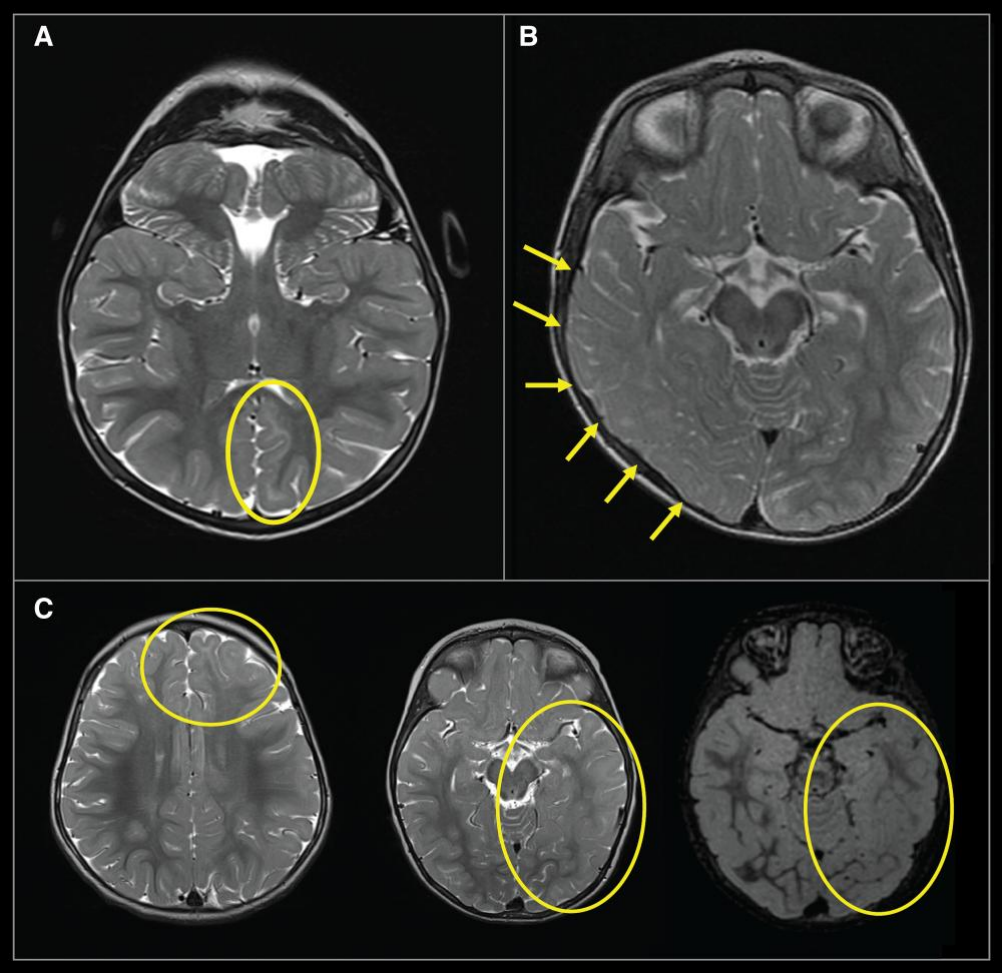
**Representative MRI scans from this cohort demonstrating features of MOGHE subtypes 1 and 2**. (**A**) Shows axial view MRI of individual AA0190-01. Evidence of increased fluid attenuated inversion recovery (FLAIR) signal at the corticomedullary junction (consistent with MOGHE subtype 1) is indicated in the yellow circled area. (**B**) Shows axial view MRI of individual AA01391-01. Arrows indicate reduced corticomedullary differentiation because of increased signal of the adjacent white matter in the right temporal lobe (MOGHE Subtype 2). (**C**) Shows three axial slices from individual AA0228-01 with features of MOGHE subtype 2 in the posterior region, and subtype 1 in anterior regions, indicated by yellow circles.

The MRI features of all 13 reclassified children were consistent with those of their revised histopathologic diagnosis. In particular, for the children with MOGHE, imaging was consistent with MOGHE subtype 1 in six children, subtype 2 in four and one child had subtype 2 posteriorly and subtype 1 anteriorly (Fig. 3).

### Final diagnostic classification and genotype-histopathology correlations

Analysis of multimodal data led to reclassification in 14 children, being the 13 children reclassified after research histopathology review (with concordant genetic findings in the 11 with a genetic diagnosis made) and the child with mosaic TSC. The integrated diagnoses were 23/59 (39%) TSC, 13/59 (22%) FCDII/HME, 12/59 (20%) FCDI/mMCD/no FCD, 9/59 (15%) MOGHE, 1/59 (2%) dysembryoplastic neuroepithelial tumour and 1/59 (2%) complex MCD.

The genetic basis was identified in 22/23 (96%) children with TSC (*TSC1*, *n* = 2; *TSC2*, *n* = 20), 11/13 (85%) children with FCDII/HME (*PIK3CA*, *n* = 3; *AKT3*, *n* = 2; *MTOR*, *n* = 2; *DEPDC5*, *n* = 2; *TSC2*, *n* = 1; *NPRL3*, *n* = 1), 3/12 (25%) children with FCDI/mMCD/no FCD (*CDKL5*, *n* = 2, both with non-specific pathology; *COL4A1* & 6p25.2del, *n* = 1, with FCDI), 9/9 (100%) individuals with MOGHE (*SLC35A2*, *n* = 9), 1/1 (100%) with dysembryoplastic neuroepithelial tumour (*FGFR1*) and 1/1 (100%) with a complex MCD (*OFD1*). Of those with a genetic diagnosis identified, somatic variants accounted for only 2/23 (9%) TSC diagnoses but 21/26 (77%) children with other diagnoses. This included 11/13 (81%) children with FCDII/HME (one of whom also had a germline variant), 9/9 (100%) with MOGHE and 1/1 (100%) with complex MCD.

## Discussion

In this comprehensive study of the genetic and histopathologic aetiologies of IESS due to focal MCD, we identified the genetic aetiology in 80% (47/59) of children and demonstrated somatic variants in *SLC35A2* as a major contributor. We showed that the types of focal MCD and their genetic basis are similar to general paediatric epilepsy surgery cohorts,^11,38,39^ with the mTORopathies (TSC and FCDII/HME) accounting for 61% of our cohort, and MOGHE for 15%. In contrast to previous studies of epilepsy surgery cohorts, which were not selected for epilepsy syndrome, we focused specifically on IESS, as advancing understanding of its aetiologies is required to expedite diagnosis and improve treatment of this devastating epilepsy.

### Integration of multimodal data is important for accurate diagnosis

Genetic testing and reanalysis of histopathology and imaging features were important in our cohort for accurate diagnosis and resulted in reclassification in nearly one-quarter.

The majority of reclassifications were in children with *SLC35A2* variants, confirmed on histopathologic review to have MOGHE, with imaging features consistent with those previously reported.^34,40^ This entity was not described until recently and our patients, like others in the literature, had been previously classified as having FCD.^16^ Some had surgery prior to the description of MOGHE and the recent children did not have Olig2 staining performed on the clinical specimens. With MOGHE, now a well-defined entity, epileptologists and neuropathologists are increasingly aware of this disorder and incorporating Olig2 staining into the clinical pathway, which should increase MOGHE diagnoses in the future.

Our results highlight the importance of using an integrated diagnostic pathway that combines genetic and neuroimaging findings in addition to histopathology, as has been recommended with the most recent revisions to FCD classification.^4^ Such a multimodal approach can facilitate a diagnosis (or presumptive diagnosis) in the event of incomplete or inconclusive information. For example, if surgery is disconnective rather than resective, tissue specimens may be limited and distant from the region of most prominent malformation, where the histopathologic abnormality may be less obvious or not represented in the tissue specimen. It would be ideal, especially in the context of emerging precision non-surgical treatments, if multimodal data could reliably distinguish the different focal MCD, particularly distinguishing between FCD and MOGHE, prior to surgery; this will be an important area of ongoing work.

### The importance of somatic mosaicism in IESS

The genetic landscape of IESS with focal MCD comprises germline and brain somatic variants, identified at similar frequency (germline variants in 57% of diagnoses, somatic in 45%) across our cohort. However, the relative contribution of somatic variants differed by pathology. As expected, most individuals with TSC had germline variants. In contrast, somatic variants were predominant in FCDII/HME and MOGHE, in 81% and 100% of children, respectively.

All but one of the somatic variants identified in brain tissue were not detected in peripheral tissue, suggesting that most mosaic variants are likely isolated to brain tissue. This finding has two important clinical implications. Firstly, current clinical genomic testing has significant limitations for genetic diagnosis in most individuals with FCDII/HME or MOGHE, given testing of peripheral tissues such as blood or saliva will likely only identify germline pathogenic variants.^41,42^ Future methods for detection of brain mosaic variants that do not require brain tissue, for example, the use of CSF liquid biopsies may improve pre-surgical diagnostic yield.^43,44^ Improving the yield of clinical genomic testing of IESS would be clinically useful for raising suspicion of a focal MCD given that focal MCD are underdiagnosed because they can be difficult to identify on brain imaging during infancy.^2,45^ Secondly, as not all infants with IESS due to focal MCD (or unknown cause) undergo epilepsy surgery and have tissue available for study, brain somatic variants may constitute a more common genetic basis of IESS than previously understood, contributing to the lower diagnostic yield of clinical genomic testing in IESS compared with other early-life epilepsy syndromes.^46^

### The genetic landscape of mTORopathies in individuals with IESS is similar to those of mTORopathies causing other epilepsies

The frequency of variants in genes causing mTORopathies in our cohort was similar to that reported in broader epilepsy surgery cohorts. Germline pathogenic variants in individuals with TSC were predominant in *TSC2*, as previously established.^41,47^ In those with FCDII/HME, we identified somatic pathogenic variants in genes spanning the full PI3K-AKT-mTOR and GATOR1 complex pathways, without a clear preponderance of one gene, highlighting that the causative gene does not necessarily predict the type of epilepsy.^48^ The range of mTOR pathway genes in our cohort may reflect the variable size of malformations in these individuals. Previous studies of FCDII cohorts have noted associations between the causative gene and the malformation size, such as the predominance of somatic *MTOR* mutations in bottom of sulcus dysplasia, and *PIK3CA* and *AKT3* variants in HME.^14,18,49^ It is unclear some people with mTORopathies present with IESS compared with others in whom focal epilepsies occur, despite sharing the same genetic causes. Factors such as brain lesion size or location, or timing of somatic mutation or gene expression may be important, given they have been shown to impact age of seizure onset.^50^

### SLC35A2-related MOGHE is a major contributor to IESS

We identified nine individuals (15% of cohort) with pathogenic variants in *SLC35A2*, all of whom had an integrated diagnosis of MOGHE. Barba *et al.*^40^, in a cohort of 47 individuals, identified ‘early epileptic encephalopathy with epileptic spasms as the predominant seizure type’ as the most common presentation of *SLC35A2*-related disorders. While we are unable to replicate this given our cohort contained only individuals with IESS, we provide compelling evidence of the importance of mosaic *SLC35A2* variants as a cause of IESS.

Consistent with the findings of Barba *et al.*, we found that VAF (Supplementary Table 1) could not reliably predict lesion size or post-surgery outcome, although we cannot exclude this being due to sampling away from the site of greatest abnormality.

### An important role for genetic testing in the epilepsy surgery work-up

The diagnostic yield in individuals with histopathologic features of FCDI, mMCD or no FCD identified was low (3/12, 25%). While FCDI (particularly FCDIA) is a well-defined pathologic entity, its causes remain largely unknown, and only one of four patients in our cohort with FCDI had a genetic cause identified. All three individuals with a genetic diagnosis had germline variants detectable in peripheral tissues. It is possible that some portion of FCDI cases have an acquired basis such as pre- or perinatal stroke, traumatic brain injury or vascular lesions.^51^ It is also possible that children without a genetic diagnosis in this study may have somatic mosaic genetic causes that are present at a VAF that is too low to be detected in the brain region used for testing.^52,53^ Alternatively, IESS with MCD in these children may have resulted from polygenic causes, deep intronic variants, repeat expansions or variation in a gene yet to be associated with MCD and epilepsy.

One individual had a dual diagnosis of *COL4A1*-related disorder and a 6p25.2 deletion with imaging features of both entities. Although a bilateral imaging abnormality was recognized prior to surgery, both the imaging and electroclinical findings were markedly asymmetric and concordant with each other. This individual underwent a left temporoparietooccipital disconnection, with resolution of a bilateral epileptic encephalopathy and reduction in seizure frequency. This case highlights that a genetic diagnosis other than an mTORopathy or *SLC35A2* does not necessarily preclude benefit from surgery, although caution and case-by-case consideration is imperative.^54^

In contrast, surgery would have likely been obviated were the genetic diagnosis identified in the other two patients, who both subsequently had pathogenic variants identified in *CDKL5*. Though the phenotypes of both individuals were not entirely typical of *CDKL5* deficiency disorder, the CDKL5 diagnosis explained the ongoing seizures, dyskinesias and delayed development.^42,43^ One had no change to seizure frequency after surgery, the other had a two-month seizure free period prior to recurrence. These cases highlight that aetiologies unlikely to benefit from surgery can present similarly to surgically-remediable epilepsies, with both children having electroclinical focal seizures (in addition to their spasms), with subtle MRI abnormalities and focal PET hypometabolism in the same region. Earlier identification of these variants could have informed clinical decisions regarding necessity of surgery in these cases.

Consistent with other recent studies, our findings demonstrate the importance of genetic testing prior to surgery in infants with IESS.^44,45^ We strongly advocate that genetic testing be performed as part of the surgical work up, as the findings can have an important bearing on clinical decision making. This may be in favour of pursuing surgery, such as in the context of a pathogenic germline *DEPDC5* variant, given high rates of seizure freedom post-epilepsy surgery reported with this and other mTOR pathway genes.^46^ Conversely, the genetic findings may favour avoiding surgery and resultant potential deficits such as hemiparesis where the surgical outcome is likely to be poor, such as in infants with *CDLK5* deficiency disorder.^42,43^ In the future, it is possible that precision therapies (medical or surgical) will be tailored to the underlying genetic cause, further demonstrating the need to develop accurate tools and methods to rapidly establish a genetic diagnosis.

### Limitations

Despite high coverage genetic investigation, a genetic cause was not identified for 20% of the cohort. It is possible that pathogenic variants in genes that have not yet been linked to epilepsy or brain abnormalities were not detected as a result of the methodology choice to utilize a panel-based analysis of the genetic data. An unbiased singleton ES approach may have assisted in identifying additional genetic causes in this cohort but also would have resulted in much more burdensome curation and higher rates of variants of uncertain significance detection. We did not perform genome sequencing (GS); it is possible that GS or combined analysis of GS and RNA-seq may increase the diagnostic yield. The possibility of non-genetic causes must also be considered, prenatal vascular insults having been proposed as a cause of FCDI.^55^ Finally, our cohort does not represent all individuals with IESS due to focal MCD, as we included only individuals who had undergone epilepsy surgery. We acknowledge that our cohort may therefore be biased towards more severely affected individuals, or pathologies and/or genes more commonly associated with more severe disease. However, given the underutilization of epilepsy surgery in those with known malformations, and the under-recognition of focal MCD in infants in particular, it is likely that some individuals with IESS due to focal MCD were not operated on despite being equally severely affected.^6^

### Conclusion and future directions

This study provides valuable insights into the aetiologic landscape of IESS with focal MCD and advances understanding of the role and limitations of genomic technologies in the diagnosis of this patient group. Somatic mosaicism is an important genetic mechanism in IESS with focal MCD and *SLC35A2*-related MOGHE is a major contributor. Our findings highlight areas of focus for future work to improve use of existing treatments, and potentially development of novel therapies, to further improve outcomes in these common causes of IESS. Understanding the histopathologic and genetic basis of IESS due to focal MCD will aid in optimizing diagnostic pathways to expedite identification of a surgically-remediable cause of IESS and in future may inform identification or development of treatment alternatives to surgery.

## Supplementary Material

fcaf034_Supplementary_Data

## Data Availability

Anonymized data created or analysed in this study will be shared by request from a qualified investigator, where consistent with participant consent. Patient level data are reported in Supplementary Table 1.
