# Supplementary material for: The genetic landscape and classification of infantile epileptic spasms syndrome requiring surgery due to suspected focal brain malformations
Source: Brain Commun. 2025 Jan 25;7(1):fcaf034. doi: 10.1093/braincomms/fcaf034 (PMC11806418; doi:10.1093/braincomms/fcaf034)
Supplement: fcaf034_Supplementary_Data [file fcaf034_supplementary_data.pdf]

Supplementary Materials

Supplementary Table 1: Clinical and genetic data on 59 children with infantile epileptic spasms syndrome who underwent epilepsy surgery.

Individual ID	Sex	Age Sz onset (months)	Sz types	Surgery			Initial pathology	Genetic findings			Discor dance	Revised pathology	MR imaging consist ent	Integrated diagnosis
				Age at surgery (years)	Surgery location	Engel class		Gene	Variant	VAF				
AA0068-01	M	9	Sp, **Fo**	8	R TPO	III	Complex MCD, Aicardi-like	*OFD1*	c.2192_2216dup; p.(P740Qfs*18)	21.0%	N	n/a	n/a	Complex MCD, Aicardi-like
AA0214-01	F	4	**Fo**, Sp	1.6, 3	R F, R P	IV	DNET	*FGFR1*	Chr8:38413607-38419737x3	Het	N	n/a	n/a	DNET
AA0221-01	F	1	**Fo**, Sp	0.8	L TPO	III	FCDI	*COL4A1*	c.634G>A; p.(G212S)	Het	N	n/a	n/a	FCDI
								TUBB2A/TUBB2B	Chr6:3155598-3224833x1	Het	N			
AA0171-01	M	2	Sp, **Fo**	3	R T	I	FCDIIA	*DEPDC5*	c.2390delA ; p.(Q797Rfs*18)	Het	N	n/a	n/a	FCDIIA
								DEPDC5	c.3994C>T ; p.(R1332*)	3.9%	N			
AA0235-01	M	5	**Fo, Sp**	0.5, 2	L I/P, L I/P	IV	FCDIIA	*DEPDC5*	c.2527C>T; p.(R843*)	Het	N	n/a	n/a	FCDIIA
AA2691-01	M	0	**Sp, To**	0.1	L H (incomplete)	I	Non-specific	*PIK3CA*	c.1624G>A; p.(E542K)	13.0%	Y	FCD IIA	Y	FCDIIA
AA0239-01	M	0	**Fo, Sp**	0.7	R H	I	Non-specific	*PIK3CA*	c.1624G>A; p.(E542K)	20.0%	Y	FCD IIA	Y	FCDIIA
AA1485-01	M	4	Sp, **Fo**	7	R F	I	FCDIIA	*TSC2*	c.336+1G>A	7.5%	N	n/a	n/a	FCDIIA
AA0097-01	M	0	**Fo**, Sp	1.4, 3	R H (incomplete), R F	I	FCDIIA/HME	*AKT3*	c.49G>A; p.(E17K)	4.9%	N	n/a	n/a	FCDIIA/HME
AA0404-01	M	0	**Fo**, Sp	1	R H	I	FCDIIA/HME	*AKT3*	c.49G>A; p.(E17K)	5.1%	N	n/a	n/a	FCDIIA/HME
AA0342-01	M	0	**Fo, Sp**	0.2	R H	I	FCDIIA/HME	*MTOR*	c.4348_4359del; p.(Y1450_L1453del)	7.4%	N	n/a	n/a	FCDIIA/HME
AA0175-01	M	0	**Fo, Sp**	0.3, 6	R TPO/R F, R H	II	FCDIIA/HME	*NPRL3*	c.1375_1376dupAC; p.(S460Pfs*20)	Het	N	n/a	n/a	FCDIIA/HME
AA0096-01	M	0	Fo, **Sp**	0.2, 0.6, 5, 9	L TPO, L TPO, L P, L TPO	I	FCDIIA/HME	*PIK3CA*	c.3140A>G; p.(H1047R)	29.0%	N	n/a	n/a	FCDIIA/HME
AA0199-01	F	15	Sp, **Fo**	2, 3	R F, R F	I	FCDIIB	*MTOR*	c.7273_7275dup; p.(P2425dup)	4.1%	N	n/a	n/a	FCDIIB
AA2561-01	M	7	**Sp**, To	3	R TPO	I	Non-specific	*SLC35A2*	c.206C>T; p.(T69I)	1.0%	Y	MOGHE	Y	MOGHE
AA0236-01	F	5	**Sp**	1.3	L TPO	II	NAD	*SLC35A2*	c.547C>T; p.(Q183*)	1.8%	Y	MOGHE	Y	MOGHE
AA0164-01	M	4	**Sp**	2, 4	R F, R H	I	Gliosis	*SLC35A2*	c.553C>T; p.(Q185*)	1.9%	Y	MOGHE	Y	MOGHE
AA0228-01	F	14	**Sp**, To	1.8, 4	L TPO, L F	I	FCDIA	*SLC35A2*	c.626_628delCCT; p.(S209del)	5.6%	Y	MOGHE	Y	MOGHE
AA1391-01	M	7	**Sp**	2	R TPO	I	NAD	*SLC35A2*	c.502C>T; p.(Q168*)	7.6%	Y	MOGHE	Y	MOGHE
AA0084-01	M	3	Sp, **Fo**	2.7, 7	L F, L F	I	FCDIIA	*SLC35A2*	c.136C>T; p.(Q46*)	20.0%	Y	MOGHE	Y	MOGHE
AA0190-01	F	2	Sp, **Fo**	6	R F	I	FCDIIA	*SLC35A2*	c.665_667delAGA; p.(K222del)	20.0%	Y	MOGHE	Y	MOGHE
AA0161-01	M	6	**Sp**, Fo	3, 7	R T, R TPO	UK	FCDIIA	*SLC35A2*	c.511T>C; p.(S171P)	35.0%	Y	MOGHE	Y	MOGHE
AA0148-01	M	16	**Sp**, To	3,4,8	L F, L F/T/I, L H	I	FCDIA	*SLC35A2*	c.359_360delTC; p.(L120Hfs*7)	41.0%	Y	MOGHE	Y	MOGHE
AA0087-01	F	2	Sp, **Fo, To**	7	L T	IV	Non-specific	*CDKL5*	c.2706delG; p.(Q902Hfs*25)	Het	N	n/a	n/a	No FCD
AA2575-01	F	9	**Fo**, **Sp**, To	2.5	L H	IV	Non-specific	*CDKL5*	ChrX:18425546-18425744x1	Het	N	n/a	n/a	No FCD
AA0151-01	F	2	**Fo**, Sp, **To**	13	R F	I	TSC	*TSC1*	c.1886delA; p.(K629Rfs*24)	20.7%	N	n/a	n/a	TSC
AA0224-01	M	6	**Fo**, Sp	2.6	R T	II	TSC	*TSC1*	c.1498C>T; p.(R500*)	Het	N	n/a	n/a	TSC
AA0936-01	F	7	**Sp**	1	R F	I	FCDIIB	*TSC2*	c.2071delC; p.(R691Afs*7)	13.0%	Y	TSC	n/a	TSC
AA0017-01	M	5	Sp, **Fo**	16	L F	IV	TSC	*TSC2*	c.4527_4529delCTT; p.(F1510del)	Het	N	n/a	n/a	TSC
AA0033-01	F	2	Sp, **Fo**	1.2, 2.9, 4, 13	L T, R TPO, R TP, R FT	III	TSC	*TSC2*	c.2251C>T; p.(R751*)	Het	N	n/a	n/a	TSC
AA0092-01	M	6	**Fo**, Sp	1.5, 2.3, 2.8, 6	R F, R FT, R F, R FC	III	TSC	*TSC2*	c.4736G>T; p.(G1579V)	Het	N	n/a	n/a	TSC
AA0093-01	F	9	Sp, **Fo**	2.5, 4.9	R FP, R T	III	TSC	*TSC2*	c.4991dupG; p.(Q1665Pfs*41)	Het	N	n/a	n/a	TSC
AA0140-01	F	4	Sp, **Fo**	3.5	R F	I	TSC	*TSC2*	c.5238_5255del; p.(H1746_R1751del)	Het	N	n/a	n/a	TSC
AA0143-01	F	6	**Sp, Fo**	2.8, 5, 10.7	L C, L O, R F	III	TSC	*TSC2*	c.4648+1G>T	Het	N	n/a	n/a	TSC
AA0145-01	F	1	**Fo**, Sp	0.9, 1.4, 7.2	L C, L T, L PT	III	TSC	*TSC2*	c.5140C>T; p.(Q1714*)	Het	N	n/a	n/a	TSC
AA0178-01	F	4	Sp, **Fo**	2.8, 7.8	L C, L O	I	TSC	*TSC2*	c.3281C>A; p.(S1094*)	Het	N	n/a	n/a	TSC
AA0187-01	M	1	**Fo, Sp**, To	0.7, 0.8, 1.1, 4.2, 4.5	L T, L O, R FT, L FC, R O and sylvian	III	TSC	*TSC2*	c.3685_3686insA; p.(E1230Gfs*4)	Het	N	n/a	n/a	TSC

AA0264-01	F	3	Sp, **Fo, To**	16	L C	III	TSC	*TSC2*	c.5024C>T; p.(P1675L)	Het	N	n/a	n/a	TSC
AA0302-01	F	0	**Fo**, Sp, **My, MA**	1.9	L P	I	TSC	*TSC2*	c.1840-1G>A	Het	N	n/a	n/a	TSC
AA0316-01	M	0	**Fo**, Sp, To, My	3.4	L F	III	TSC	*TSC2*	c.724_725insG; p.(T242Sfs*96)	Het	N	n/a	n/a	TSC
AA0329-01	F	0	**Fo**, Sp	2, 3.5, 5.3	R F/I, R T, R F	I	TSC	*TSC2*	c.5227C>T; p.(R1743T)	Het	N	n/a	n/a	TSC
AA0938-01	F	4	**Sp, Fo, My,** AA, At	0.6, 2.3, 3.7	R T, L PO, L C	III	TSC	*TSC2*	c.4573C>T; p.(Q1525*)	Het	N	n/a	n/a	TSC
AA1408-01	M	11	**Fo**, **Sp**	2, 2.2	L T, R C	I	TSC	*TSC2*	c.3574delC; p.(Q1192Rfs*18)	Het	N	n/a	n/a	TSC
AA0166-01	M	8	**Sp**, **Fo**	4.2, 5.5, 9.4	L F, R F, L F	IV	TSC	*TSC2*	Chr16:2062961-2086850x1	Het	N	n/a	n/a	TSC
AA0313-01	F	4	**Fo**, Sp	2, 2.5, 4.4, 6.3	L FT, L F, R F, R P	IV	TSC	*TSC2*	Chr16:2090008-2100467x1	Het	N	n/a	n/a	TSC
AA0195-01	M	3	**Fo**, Sp	2.4, 4.8, 5.3	R FP, L FT, L F	I	TSC	*TSC2*	Chr16:2134591-2136455x1	Het	N	n/a	n/a	TSC
AA1027-01	M	6	**Sp, Fo**	5.3	R PO	I	TSC	*TSC2*	Chr16:2046127-2050488x1	Het	N	n/a	n/a	TSC
AA0327-01	M	11	**Sp**	1.5	L TPO	I	FCDI	...			N	n/a	n/a	FCDI
AA0265-01	M	4	**Sp**	4	R F	I	FCDIIA	...			Y	FCD I	Y	FCD I
AA0294-01	F	6	**Sp**	3	L H	I	FCDIIA	...			Y	FCD I	Y	FCD I
AA0193-01	F	4	**Sp**, My, At	2	L F	I	FCDIIA	...			N	n/a	n/a	FCDIIA
AA0192-01	F	1	Sp, **Fo**	1.2, 4	R F-P-I, R F	II	FCDIIA/HME	...			N	n/a	n/a	FCDIIA/HME
AA0937-01	F	6	**Sp**, To	3	R TPO	IV	Gliosis	...			N	n/a	n/a	Gliosis
AA2569-01	M	1	**Fo**, Sp, My	3, 4	R H, R F	IV	Gliosis	...			N	n/a	n/a	Gliosis
AA0203-01	M	9	**Sp, To**	3	L TPO	I	NAD	...			N	n/a	n/a	No FCD
AA0099-01	F	4	**Fo, Sp**, To	11	L T	III	Non-specific	...			N	n/a	n/a	No FCD
AA0215-01	F	6	**Sp, Fo**	2, 4	L F, L F-C	IV	Non-specific	...			N	n/a	n/a	No FCD
AA2537-01	M	11	**Sp**	1.6	R H	I	Non-specific	...			N	n/a	n/a	No FCD
AA0142-01	M	9	**Sp Fo, My, To**	3.8	R FT	IV	TSC	...			N	n/a	n/a	TSC

AA, atypical absence seizures; At, atonic seizures; C, cortical; F, frontal; FCD, focal cortical dysplasia; Fo, focal seizures; H, hemispheric disconnection; Het, heterozygous; HME, hemimegalencephaly; I, inferior; L, left; MOGHE, mild malformation of cortical development with oligodendroglial hyperplasia in epilepsy; MA, myoclonic-atonic seizures; My, myoclonic seizures; N, no; NAD, no abnormality detected; No FCD, no definite FCD on histopathology; P, parietal; R, right; Sp, spasms; Sz, seizure; T, temporal; To, tonic seizures; TPO, temporal-parietal-occipital; TSC, tuberous sclerosis complex; VAF, variant allele frequency; Y, yes;. Seizure types listed in order of onset. Indication for surgery is shown in bold.
